# Role of L-Arginine in the Gut–Liver Axis of Female Mice: Mediating Ethanol’s Alterations in Hepatic Steatosis and Oxidative Stress

**DOI:** 10.3390/antiox15050537

**Published:** 2026-04-24

**Authors:** Kaitlyn Daff, Yingchun Han, Zhuoying Feng, Mala Upadhyay, Vyshnavi Sivampeta, Abirami Rajasekaran, Naseer Sangwan, Gail A. M. Cresci

**Affiliations:** 1 Department of Microbial Sciences in Health, Cleveland Clinic Research, Cleveland, OH 44195, USAsangwan@ccf.org (N.S.); 2 Department of Nutrition, Case Western Reserve University, Cleveland, OH 44106, USA; 3 Lerner College of Medicine of Case Western Reserve University, Cleveland, OH 44195, USA; 4 Department of Heart, Blood and Kidney Research, Cleveland Clinic Research, Cleveland, OH 44195, USA; 5 Microbial Sequencing & Analytics Resource (MSAAR) Facility, Shared Laboratory Resources (SLR), Cleveland Clinic Research, Cleveland, OH 44195, USA; 6 Department of Gastroenterology, Hepatology and Nutrition, Cleveland Clinic, Cleveland, OH 44195, USA

**Keywords:** gut–liver axis, gut microbiome, arginine, ethanol, oxidative stress, hepatic steatosis, body composition

## Abstract

Ethanol exposure causes microbial imbalance, damages the gut barrier, and increases oxidative stress along the gut–liver axis, leading to the development and progression of alcohol-associated liver disease (ALD). Arginine is a conditionally essential amino acid that may play a key role in maintaining redox homeostasis and mediating host–microbiota crosstalk. We hypothesized that supplemental arginine provided during chronic ethanol exposure in mice would mitigate oxidative damage via the gut–liver axis. Our findings suggest that arginine supplementation mediated hepatic steatosis, preserved body weight and fat, and reduced oxidative stress in the gut–liver axis. These changes were associated with alterations in gut microbiota composition and function. These data support a potential role for arginine supplementation in mitigating ethanol-induced oxidative damage via the gut–liver axis.

## 1. Introduction

Disruption in the inter-organ crosstalk between the gut and liver, known as the gut–liver axis, occurs during the development of alcohol-associated liver disease (ALD) [1]. Chronic ethanol consumption alters the composition and function of the gut microbiome, a condition termed gut dysbiosis, which may contribute to the development of ALD [2]. Ethanol-induced gut dysbiosis can disrupt host metabolism and redox homeostasis, leading to oxidative damage through increased reactive oxygen species and lipid peroxidation [3,4,5].

Chronic ethanol exposure alters nutrient absorption, further disrupting tissue homeostasis as ALD progresses. Amino acid uptake and transport are dysregulated during ethanol consumption [6]. Arginine is a conditionally essential amino acid with unique significance in ALD. A growing body of evidence suggests that arginine metabolism and tissue uptake are altered during the progression of ALD [7]. Arginine can be exogenously acquired through the diet or endogenously synthesized by intestinal cells or produced by the gut microbiota [8]. It plays a vital role in maintaining redox balance, supplying substrates for antioxidant defenses, and affecting lipid metabolism during liver damage [9]. How supplemental arginine may impact the gut–liver axis and gut microbiome during chronic ethanol exposure is not fully understood. Due to arginine’s role in maintaining redox homeostasis, here we evaluated the role of supplemental arginine in ethanol-induced oxidative stress and disruption in the gut–liver axis.

## 2. Materials and Methods

### 2.1. Chronic Ethanol Feeding Model

The Lieber–DeCarli chronic feeding model [10,11] was used to examine the gut–liver axis during ethanol exposure. C57BL/6 female mice (8–10 weeks) were purchased from Jackson Laboratory (Bar Harbor, ME, USA) and randomized to receive the ethanol-containing liquid diet ± L-arginine added directly to the diet. Control mice were fed an isocaloric liquid diet containing maltose dextrin substituted for ethanol ± L-arginine added directly to the diet. There were four total treatment groups: pair-fed control, pair-fed + arginine supplementation, ethanol-only fed, and ethanol-fed + arginine supplementation. The total number of mice used for each analysis is described in each figure legend.

Mice were acclimated to a liquid diet using the control liquid diet for 3 days before following the previously established chronic ethanol feeding model [10]. In brief, mice were allowed free access to an ethanol-containing liquid diet with increasing concentrations of ethanol over 25 days: 1% and 2% volume/volume (*v*/*v*) ethanol for two days each, 4% *v*/*v* ethanol for seven days, 5% *v*/*v* ethanol for seven days, and 6% *v*/*v* ethanol for seven days before euthanasia on day 25 of ethanol exposure. Pair-fed control and pair-fed arginine mice were fed the same volume of liquid diet as their weight-matched ethanol cohort to ensure that mice receive isocaloric diets and that there were no differences in nutrient intake aside from ethanol between groups.

### 2.2. Arginine Supplementation

The Lieber–DeCarli liquid diet provides a baseline arginine concentration of 1.3 mg/mL (Supplementary Materials). On average, this is equivalent to 0.8 mg of arginine per gram of body weight. Previous research shows arginine intake of approximately 2.5 mg/g of body weight may help reduce lipid peroxidation and innate immune activation [9,12]. Therefore, in addition to the arginine included in the Lieber–DeCarli diet, additional L-Arginine (Sigma-Aldrich, Milwaukee, WI, USA) was added directly to the liquid diet to provide a total of 2.5 mg/g body weight/day (4.9 mg/mL) throughout the feeding period.

### 2.3. Body Composition Analysis

The mice had their total body weight measured three times per week throughout the study using a digital scale. To assess if ethanol consumption altered overall body composition, ECHO-MRI imaging (Echo Medical Systems, Houston, TX, USA) was used to measure changes in fat mass and fat-free mass (FFM) at baseline and 24 h before euthanasia [13].

### 2.4. Liver Triglyceride Analysis

Following euthanasia, liver tissue was collected, flash-frozen, and stored at −80 °C. To determine liver triglyceride (TG) levels, the Pointe Triglyceride Reagent set was used (MedTest, Canton, MI, USA). In short, glycerol phosphate oxidase (GPO) enzymatic activity is measured to assess the presence of TG in tissue. GPO catalyzes the hydrolysis of glycerol-1-phosphate and produces hydrogen peroxide. This product can then react with horseradish peroxidase to produce a fluorescent product that is directly proportional to the concentration of TG in the sample. To perform this assay, 80–85 mg of liver tissue was weighed and digested using a buffer containing H_2_O, 100% EtOH, and KOH. Digestion was carried out by heating the tissue at 70 °C with intermittent vortexing for homogenization. After the tissue was fully digested and cooled to room temperature, 2M Tris-HCl (pH 7.5) was added to dilute the samples to a 1:5 ratio. Glycerol phosphate oxidase (GPO) reagent was then added to the diluted samples, and the plates were read at a 500 nm wavelength. Raw data were exported to Excel and converted into mg per mg liver tissue. TG levels were normalized to total liver tissue weight (mg), total body weight (g), and fat-free mass (g) for statistical analysis and graphical representation.

### 2.5. Cecal Shotgun Metagenomics Sequencing, Bioinformatics, and Statistical Analysis

Following euthanasia, cecum samples were dissected, flash-frozen, and stored at −80 °C until analysis. DNA was extracted using the Zymo Quick-DNA Fecal/Soil Microbe MiniPrep Kit (Zymo Research, Irvine, CA, USA) according to the manufacturer’s instructions. Shotgun metagenomic libraries were prepared using the QIAseq FX DNA Library Kit (QIAGEN, Germantown, MD, USA) following previously published protocols [14]. In summary, 150 ng of input DNA was fragmented to an average size of 350 bp using a Covaris M220 focused ultrasonicator (Covaris, Woburn, MA, USA). The fragmented DNA was then purified with the QIAseq FX Bead kit at a 1.8× ratio. End-repair, A-tailing, and adapter ligation were performed using the QIAseq FX Enzyme Mix. Multiplexing was carried out with QIAseq Unique Dual Index Adapters (QIAGEN). Ligated products were cleaned with QIAseq FX Beads at a 0.8× ratio. Libraries were enriched through PCR with reactions containing 25 µL of QIAseq HiFi Master Mix, 5 µL of QIAseq IL-Index Primers, and 15 µL of ligated DNA, for a total volume of 50 µL per reaction. Cycling conditions included 1 cycle at 95 °C for 2 min, followed by 8–12 cycles of 98 °C for 20 s and 65 °C for 30 s, ending with a final extension at 72 °C for 1 min. Finally, size selection was performed using the dual QIAseq FX Bead Cleanup kit to retain fragments between 300 and 700 bp.

Libraries were quantified using the Qubit dsDNA HS Assay, and their size distribution was assessed on a Bioanalyzer High Sensitivity DNA chip. Using an Illumina NovaSeq 6000 S4 flow cell, 1.3 µL of the final cleaned DNA pool was loaded. Sequencing targeted approximately 15 million read pairs per sample for 2 × 150 bp paired-end sequencing with an S4 Reagent Kit (300 cycles).

Following paired-end sequencing, raw FASTQ quality was assessed using FastQC v0.11.9. Low-quality bases (Phred < 20) were trimmed with Trimmomatic v0. Then, contaminant host reads were removed by mapping against the reference genome using Bowtie2 v2.4.4. Taxonomic classification was performed with Kraken2 v2.1.211, utilizing a custom database consisting of RefSeq bacterial, archaeal, viral, and fungal genomes. Per-sample taxonomic abundance was generated with parameters set to a confidence threshold of 0.1, a read length of 150 bp, and a recalculated k-mer distribution for our database. Finally, the results were summarized at the genus and species level and converted to raw counts for downstream analysis.

### 2.6. Real-Time Quantitative Polymerase Chain Reaction (RT-qPCR)

Following euthanasia, intestinal tissue from the jejunum and proximal colon was dissected, immediately preserved in RNALater, and stored at −20 °C until RNA extraction. An RNAeasy Plus Universal Mini Kit (Qiagen, Germantown, MD, USA) was used for tissue RNA extraction according to the manufacturer’s instructions. RNA quantification was performed using a Nanodrop ND1000 Spectrophotometer (Thermo Fisher Scientific, Waltham, MA, USA). To prepare cDNA, 2 µg of prepared RNA was reverse-transcribed using SuperScript IV VILO (Invitrogen, Carlsbad, CA, USA). RT-qPCR was performed using QuantStudio 5 (Applied Biosciences, Carlsbad, CA, USA). PowerUp SYBR Green was used as the fluorescent dye to report mRNA amplification. To identify targets of interest, 1 µM of primers (Table 1) was used. The Comparative Threshold (CT) method was used to determine the relative mRNA expression of genes of interest. Glyceraldehyde 3-phosphate (GAPDH) was used as the housekeeping gene [15].

### 2.7. Western Blotting of Liver Tissue

Following euthanasia, the liver was dissected, flash-frozen in liquid nitrogen, and stored at −80 °C until protein analysis. To prepare liver lysates, tissue was suspended in lysis buffer containing 1% Triton X-100, 50 mM Tris-HCl, 150 mM NaCl, 1 mM EDTA, 0.1% Na-deoxycholate, and 0.1% SDS, with Pierce Protease and Phosphatase Mini Tablets (ThermoFisher, Waltham, MA, USA). The tissue was then homogenized by sonication, and the protein lysate concentration was measured using the Pierce BCA Protein Assay Kit (ThermoScientific, Waltham, MA, USA), following the manufacturer’s instructions. Polyvinylidene Fluoride membranes were used to probe for CYP2E1, 4-HNE, ANGPTL4, LPL, LCN2, PARP HSC70, and Beta-actin protein expression (Table 2). Protein expression was visualized with enhanced chemiluminescence, and images were captured using Image Lab Touch Software on a ChemiDoc MP Imaging System (Bio-Rad). Densitometry analysis of protein expression was performed with ImageJ software v. 1.54i (National Institutes of Health, NIH, Bethesda, MS, USA).

### 2.8. Immunohistochemical Staining of Intestinal Tissue

Intestine tissue (jejunum) was embedded in Tissue-Tek O.C.T (optimal cutting temperature) Compound (4583; Sakura Finetek, Tokyo, Japan) and frozen. Then, 10 µm sections were cut using Leica CM 1950 cryostat. For labeling, sections were washed, blocked in 1XPBS supplemented with 10% donkey serum for 60 min, and incubated with primary antibodies overnight at 4 °C. The next day, sections were incubated in secondary antibodies coupled to Alexa 488 (1:500 dilution). Nuclei were labeled with DAPI (NB2-31156, Novus Biologicals LLC, Centennial, CO, USA). The antibodies used included anti-ZO-1 (1:500; rabbit; cat# 21773-1-AP; Proteintech Group Inc., Rosemont, IL, USA) and anti-E-cadherin (10 µg/mL; rat; cat#13-1900; ThermoFisher Scientific, Rockford, IL, USA). Once stained, sections were mounted in Fluoromount-G mounting media (Cat# 17984-25, Electron Microscopy Sciences, Morgantown, PA, USA). Images were acquired using an inverted fluorescence microscope (Model BZ-X810, Keyence Corporation of America, Itasca, IL, USA). At least three images per tissue section were acquired for four to six mice per experimental condition. All images were acquired using the same acquisition parameters. The quantification of positive staining was performed using ImagePro plus 7.0 software (Media Cybernatics, Silver Spring, MD, USA).

### 2.9. Statistical Analysis

Power calculations were performed using the following website: http://powerandsamplesize.com/Calculators/ (accessed on 29 Ocotber 2024). Power calculation based on the primary endpoint of total body weight change (g) indicates that six mice per group will be required to determine statistical significance at 80% power with *p* < 0.05.

GraphPad Prism^®^ 10 ver. 10.1.2 (GraphPad, San Diego, CA, USA) was used for statistical analysis. The Shapiro–Wilk test was used to assess the normality of all data. Normally distributed data were analyzed using a two-tailed Student’s *t*-test. Data that were not normally distributed were analyzed using Welch’s *t*-test. The significance threshold was set at *p* < 0.05. Outliers were identified using the robust nonlinear regression method (ROUT) and excluded when indicated [16]. All data are presented as mean values ± standard deviation.

## 3. Results

### 3.1. Arginine Supplementation Protected Against Body Weight and Fat Mass Losses During Chronic Ethanol Feeding

**Figure 1:**
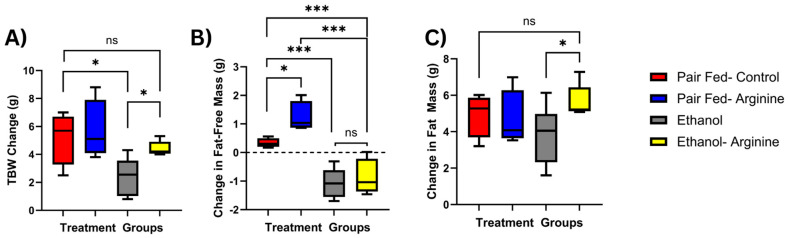
Effects of chronic ethanol feeding ± arginine on body composition. Mice were exposed to chronic feeding with the Lieber–DeCarli diet ± ethanol ± arginine. Before treatment, baseline measurements of total body weight (TBW), total body fat-free mass (FFM), and total body fat mass were measured using an electronic scale and ECHO-MRI, respectively, and repeated 24 h before euthanasia. Total body composition changes were calculated and compared. (**A**) TBW change (g). (**B**) Change in FFM (g). (**C**) Change in total body fat mass. ns: not significant, * *p* < 0.05, *** *p*< 0.001. Total mice represented by box plots: Pair fed-control *n* = 8, Pair-fed-arginine *n* = 8, Ethanol *n* = 12, Ethanol– Arginine *n* = 9.

During the 25-day feeding period, mice are expected to experience moderate weight gain. Change in total body weight (TBW) was calculated using the following formula: Final body weight (g) − Initial body weight (g). On average, pair-fed control mice gained 6 g during this period (Figure 1A), and pair-fed arginine mice experienced a similar weight gain. However, weight gain in only ethanol-fed mice was significantly lower, averaging only 2 g during the feeding period (*p* = 0.0251). Compared to pair-fed control mice, ethanol–arginine mice experienced similar weight gain, and these mice had a significantly lower TBW change than the only ethanol-fed mice (*p* = 0.012).

Body composition was measured using ECHO MRI, and changes to Fat Free Mass (FFM) were calculated using the following formula: Final FFM (g) − Initial FFM (g). Ethanol exposure significantly decreased FFM compared to pair-fed mice, regardless of arginine supplementation (Figure 1B; *p* = 0.0029). Changes to total body fat were calculated using the following formula: Final fat mass (g) − Initial fat mass (g). When measuring total body fat, although not significant, only ethanol-fed mice had lower body fat than pair-fed control mice, and only ethanol-fed mice had significantly lower fat mass than ethanol + arginine mice (Figure 1C, *p* = 0.0055). There were no differences in fat mass between ethanol-fed arginine mice and either of the pair-fed groups, suggesting that the similar total body weight change observed in these groups is likely due to a consistent rate of fat mass gain during the feeding period.

### 3.2. Arginine Supplementation During Chronic Ethanol Feeding Altered Hepatic Steatosis and Markers of Oxidative Stress

**Figure 2:**
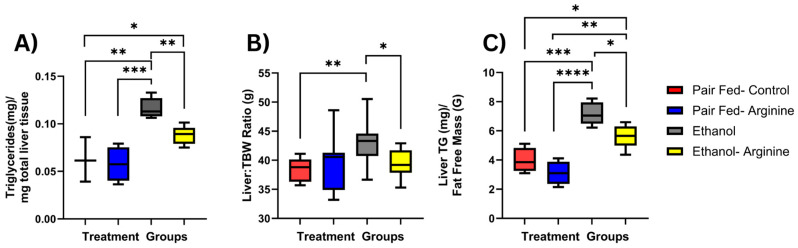
Arginine supplementation reduced chronic ethanol-induced liver triglyceride accumulation. Mice were exposed to the 25-day Lieber–DeCarli chronic ethanol feeding model, and following euthanasia, the liver was dissected and triglyceride (TG) concentrations were assessed. (**A**) TG levels (mg/mg liver tissue) were normalized to total liver tissue wt (g). (**B**) Total liver tissue weight (mg) was normalized to total body weight (g) at euthanasia. (**C**) Liver TG levels were normalized to final FFM (g). * *p* < 0.05, ** *p* < 0.01, *** *p* < 0.005, **** *p*<0.0001. Box plots represent the following mice per group: Pair fed-control *n* = 7, pair fed-arginine *n* = 8, ethanol *n* = 11, ethanol–arginine *n* = 9.

Hepatic steatosis is a key early feature of ALD. Chronic ethanol exposure elevates lipid deposition through several mechanisms. It increases fatty acid uptake into hepatocytes, ramping up hepatic de novo lipogenesis while decreasing fatty acid β-oxidation. Together, this increases the liver’s fatty acid pool and favors lipogenesis and hepatic triglyceride accumulation [17,18]. To evaluate hepatic steatosis in our model, we measured liver triglyceride (TG) levels, and as expected, ethanol-exposed mice had significantly higher liver TG levels compared to pair-fed controls when normalized to total liver weight (*p* = 0.0006; Figure 2A), TBW (*p* = 0.0044; Figure 2B), and FFM (*p* = 0.0003; Figure 2C). Ethanol-fed-arginine mice showed significantly lower hepatic TG levels compared to ethanol-fed control mice when normalized to liver weight, TBW, and FFM (*p* = 0.0111; Figure 2C). Overall, this suggests that arginine supplementation may protect against ethanol-induced hepatic triglyceride accumulation.

**Figure 3:**
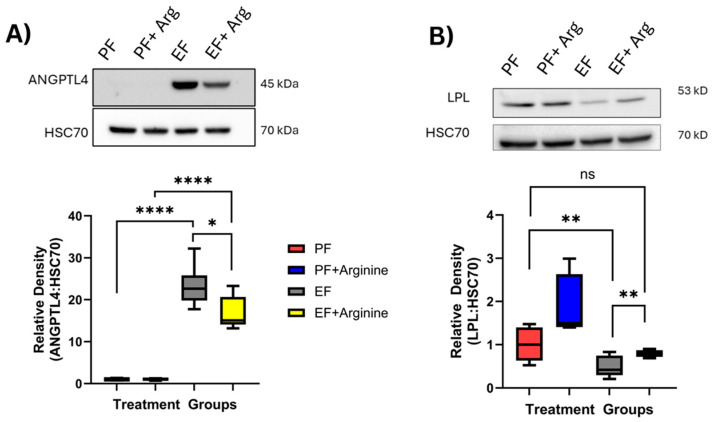
Arginine supplementation mediated hepatic lipid metabolism. Following the 25-day Lieber–DeCarli chronic ethanol feeding model described in Figure 1, mice were euthanized, and the liver was dissected and used to assess protein expression of angiopoietin-like protein 4 (ANGPTL4) and lipoprotein lipase (LPL) via Western blotting. (**A**) Bands for ANGPTL4 were visualized at 45 kDa and normalized to the expression of HSC70. Representative bands are shown. Densitometry analysis of ANGPTL4: HSC70 protein expression. Box plots represent the following number of mice per group: Pair fed-control = 6, pair fed-arginine = 4, ethanol = 9, ethanol–arginine = 6. (**B**) Bands for LPL were visualized at 53 kDa and normalized to the expression of HSC70. Representative bands shown. Densitometry analysis of LPL: HSC70 protein expression. ns: not significant, * *p* < 0.05, ** *p* < 0.01, **** *p* < 0.0001. Box plots represent the following number of mice per group: Pair fed-control *n* = 8, pair fed-arginine *n* = 4, ethanol *n* = 12, ethanol–arginine *n* = 5.

Experimental models have shown L-arginine supplementation to alleviate liver injury [19]. Angiopoietin-like protein 4 (ANGPTL4) is a protein expressed in the liver and adipose tissue. ANGPTL4 acts as a regulator of lipid metabolism by inhibiting lipoprotein lipase (LPL), which increases TG levels [20]. As both arginine and ANGPTL4 are involved in reducing lipid-related liver damage, we assessed whether the changes we observed in hepatic TG deposition were associated with changes in ANGPTL4 protein expression in the liver. Chronic ethanol exposure significantly increased ANGPTL4 protein expression compared to pair-fed mice (*p* ≤ 0.0001; Figure 3A). Interestingly, ethanol-fed arginine mice had significantly lower hepatic ANGPTL4 levels than ethanol-only-fed mice (*p* = 0.0253; Figure 3A).

ANGPTL4 is one of the main regulators of LPL expression; therefore, we assessed for LPL protein expression in liver tissue [21]. Chronic ethanol exposure decreased LPL expression when compared to pair-fed mice (*p* = 0.0049; Figure 3B). Ethanol-fed-arginine mice had significantly increased hepatic LPL expression compared to only ethanol-fed mice (*p* = 0.0011; Figure 3B). Additionally, hepatic LPL expression was increased in pair-fed + arginine mice compared to pair-fed control mice (*p* = 0.0246; Figure 3B).

**Figure 4:**
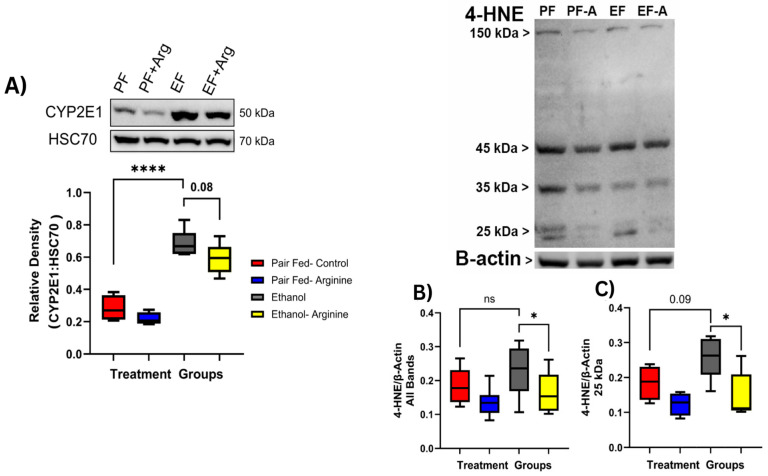
Chronic ethanol feeding influences hepatic oxidative metabolism and lipid peroxidation. Mice were exposed to chronic feeding with the Lieber–DeCarli diet ± ethanol ± arginine for 25 days. Following euthanasia, the liver was collected, and protein lysates were prepared for Western blotting as described in Section 2.6. (**A**) Bands for CYP2E1 were visualized at 50 kDa and normalized to the expression of HSC70. Representative bands shown. Densitometry analysis of CYP2E1:HSC70 protein expression. Box plots represent the following mice per group: Pair fed-control n= 4, pair fed-arginine *n* = 4, ethanol *n* = 6, ethanol-arginine *n* = 5. (**B**) Full blot showing representative multiple bands for 4-HNE protein adducts identified at multiple molecular weights. (**C**) Densitometry analysis of 4-HNE bands visualized at 25 kDa. *n* = 4–6 mice per group. ns: not significant, * *p* < 0.05; **** *p* < 0.0001. Box plots represent the following mice per group: Pair fed-control *n* = 4, pair fed-arginine *n* = 4, ethanol *n* = 6, ethanol–arginine *n* = 5.

The production of ROS has previously been characterized as a main feature of the development of alcohol-associated liver disease [22]. Oxidative damage in the liver can result from ethanol metabolism through the Microsomal Ethanol Oxidizing System (MEOS) [23]. This pathway converts ethanol into reactive oxygen species (ROS) via the enzyme Cytochrome P450 2E1 (CYP2E1). This process alters the hepatic NADH/NAD+ ratio, disrupting redox balance and leading to increased oxidative damage [22,23,24]. Thus, elevated hepatic CYP2E1 activity indicates a higher risk of oxidative injury. We assessed for CYP2E1 protein expression in liver tissue via Western blotting and found that CYP2E1 expression was significantly increased in both ethanol-fed groups compared to pair-fed control mice (Figure 4A, *p* ≤ 0.0001). Arginine supplementation did not mitigate ethanol-induced CYP2E1 expression.

Reactions between the double-carbon bonds in fatty acids within the liver and the presence of ROS result in lipid peroxidation [25]. This can lead to the production of toxic aldehyde by-products such as 4-Hydroxynonenal (4-HNE) [26]. To identify if liver TG levels were associated with increased 4-HNE adducts during chronic ethanol feeding, Western blotting of 4-HNE-bound proteins in liver tissue was performed. Upon imaging, 4-HNE adducts were observed at multiple molecular weights, including 150 kDa, 45 kDa, 35 kDa, and 25 kDa. When bands at each of these molecular weights were measured and cumulatively compared, 4-HNE was increased, but not significantly, between pair-fed controls and only ethanol-fed mice. (Figure 4B) However, when comparing all 4-HNE adducts between the ethanol-fed and ethanol-fed + arginine groups, combined 4-HNE adducts were significantly lower in ethanol-fed + arginine mice (Figure 4B, *p* = 0.0240). When bands were analyzed separately, 4-HNE adducts at 25 kDa showed the same expression pattern as the combined bands, and ethanol-fed + arginine mice had significantly reduced expression compared to only ethanol-fed mice (Figure 4C, *p*= 0.0238). This suggests that lipid peroxidation assessed by 4-HNE adduct formation may be partially mediated by arginine supplementation during chronic ethanol exposure.

Lipocalin 2 (LCN2) within the liver can also participate in the regulation of lipid metabolism and promote oxidative damage in hepatocytes via increased neutrophil infiltration into hepatic tissues [27]. However, when we assessed for LCN2 protein expression, there were no changes in hepatic LCN2 protein expression between mouse treatment groups (Supplementary Data Figure S1). CYP2E1 induction elevates ROS, which can induce DNA damage and activate Poly(ADP-ribose) polymerase-1 (PARP-1), leading to Kupffer cell activation, hepatic inflammation, steatosis, and apoptosis [28]. We assessed PARP-1 activation by Western blotting, but there were no differences in protein expression between groups (Supplementary Data Figure S1). Together, these data suggest that ethanol induced oxidative stress, but not to the extent of causing hepatocyte cell death.

### 3.3. Arginine Supplementation Altered Bacterial Taxa and Function Within the Murine Gut

Previous work has demonstrated that chronic ethanol exposure leads to gut dysbiosis, resulting in an expansion of potentially harmful microbial communities and a reduction in bacteria that produce beneficial microbial metabolites [29]. These changes in microbial taxa can adversely affect host–microbial crosstalk and increase reactive oxygen species (ROS) production [30,31]. The accumulation of ROS can disrupt redox balance in the intestinal environment and promote the progression of ALD via the gut–liver axis [5,32].

#### 3.3.1. Bacterial Composition

**Figure 5:**
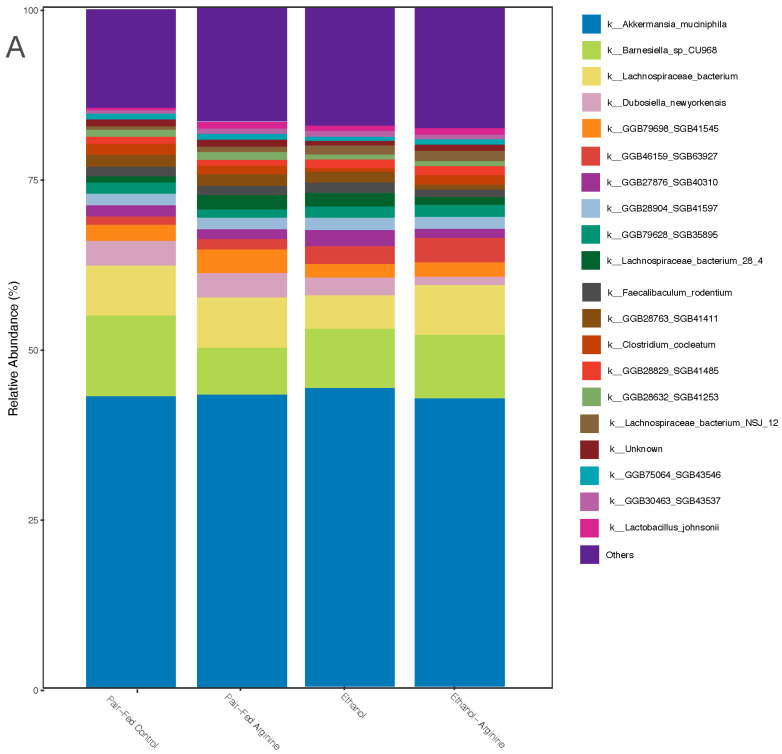

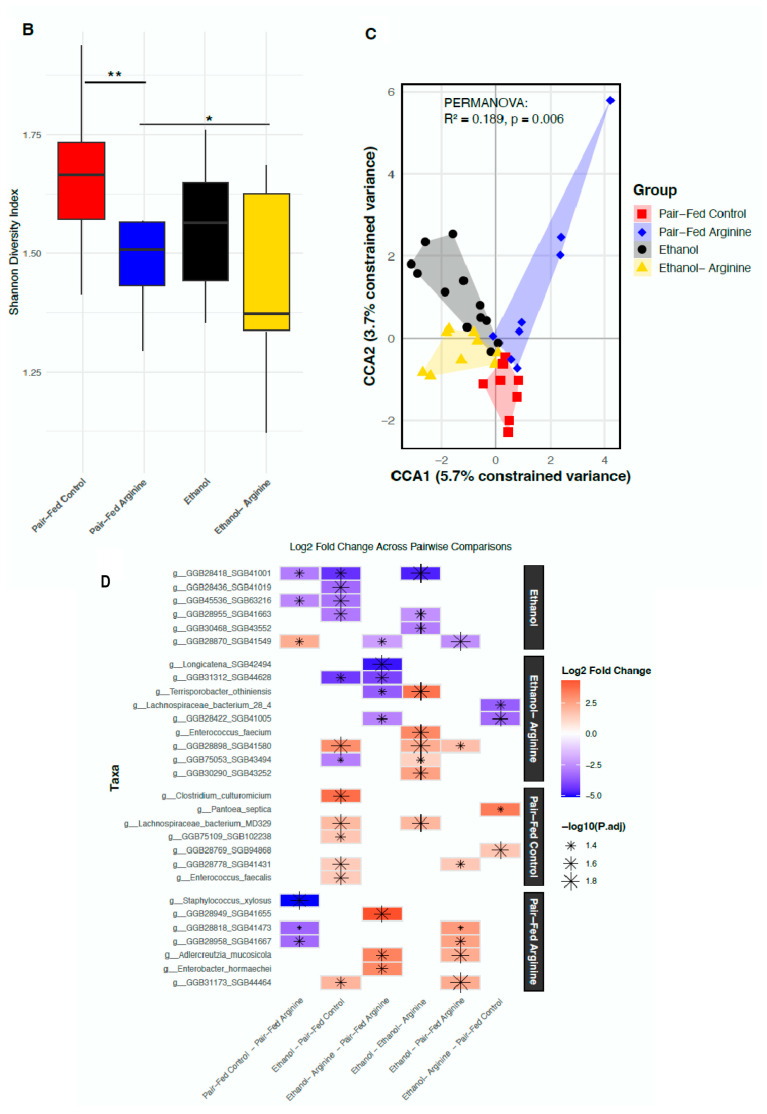
Effects of chronic ethanol feeding ± arginine on bacterial composition of cecal microbiota. Following the Lieber–DeCarli chronic ethanol feeding model, the cecum was dissected, cecal contents were isolated, and gDNA was extracted using a Zymo Quick-DNA Fecal/Soil Microbe MiniPrep Kit according to the manufacturer’s instructions. (**A**) Microbial community dynamics at the taxonomy level are shown by relative abundance indices. (**B**) Shannon Diversity Index is shown among treatment groups, with box plots representing the interquartile range (IQR) and median values. Black asterisks indicate significant pairwise differences (* *p* < 0.05, ** *p* < 0.01), as determined by Wilcoxon rank-sum tests. (**C**) Beta-diversity is presented by a CCA plot, illustrating the first two canonical components (CC1 and CC2) that explain a significant proportion of the variance in the dataset, with samples colored by treatment group (Ethanol: black, Ethanol-Arginine: yellow, Pair-Fed Control: red Pair-Fed Arginine: blue) and shapes representing distinct sample groups. Shaded convex hulls enclose the data points of each group, highlighting group transitions based on CCA scores. (**D**) Top microbial taxa are shown by a clustered differential abundance tile plot showing log2 fold changes (logFC) of taxa across pairwise comparisons, where colors denote the direction and magnitude of differential abundance (blue for decrease, red for increase, and white for no change). The size of the black points represents significance, with larger points indicating higher significance with lower adjusted *p*-values (*p* < 0.05). Taxa are ordered by maximum absolute logFC for clustering effects, with facet annotations indicating corresponding groups for each taxon. All sequencing experiments represent the following number of mice per group: Pair fed-control *n* = 8, Pair-fed-arginine *n* = 8, Ethanol *n* = 12, Ethanol- Arginine *n* = 9.

As gut microbes utilize dietary substrates to support their composition and function, we performed shotgun sequencing of bacterial gDNA isolated from cecal contents to identify microbial community dynamics during arginine supplementation with or without chronic ethanol exposure in mice. Relative abundance analysis identified bacterial taxa that influence diversity across treatment groups. Regardless of the treatment, the murine microbiome was dominated by *Akkermansia muciniphila* (Figure 5A). This commensal Gram-positive bacterium plays an essential role in producing acetate and propionate from mucins throughout the gastrointestinal (GI) tract, providing substrates for other microbes and the host [33].

Alpha-diversity is a measurement of intraindividual richness and evenness of the gut microbiome. Alpha diversity was calculated using the Shannon Diversity Index for each treatment group and compared. Arginine supplementation decreased alpha-diversity, as shown by a significantly lower Shannon Index in the pair-fed + arginine and ethanol + arginine groups compared to their respective matched groups (Figure 5B). Each treatment group displayed a unique microbial composition, as shown by Beta-diversity analysis (Figure 5C; PERMANOVA, *p* = 0.006).

To identify the bacterial species that drive diversity across treatment groups, a clustered differential abundance analysis was performed. Maximum absolute log2 fold changes (logFC) were calculated to identify top taxa clustered in each treatment condition (Figure 5D). Within the pair-fed control mice, *Clostridium culturomicium*, *Lachnospiraceae bacterium* MD329, and *Enterococcus faecalis* were the most prominent taxa with significantly higher abundances in ethanol-treated mice compared to the pair-fed controls (Figure 5D). All of these bacteria are Gram-positive members of the Firmicutes phylum.

When mice received the pair-diet with supplemental arginine, the top clustered taxa included *Staphylococcus xylosus*, *Aldercreutzia mucosicola*, and *Enterobacter hormaechei*. *S. xylosus* was significantly different in abundance between the pair-fed control and pair-fed arginine mice, with a log fold change of 1.8 (P.adj) (Figure 5D). This indicates that the overabundance of these taxa in the pair-fed-arginine group may be a key factor driving the differences between these two groups. Genomic profiling of *S. xylosus* shows that this species can express genes for both L-arginine biosynthesis and degradation, suggesting that arginine supplementation may promote the expansion of this bacterium [34].

Within the ethanol and arginine treatment cluster, *Longicatena*_SGB42494, *Terrisporobacter othenensis, Lachnospiraceae bacterium*_28, and *Enterococcus faecium* were identified as driving the gut community. These bacteria are all Gram-positive members of the Firmicutes phylum [35]. Of these bacteria, *Longicatena*_SGB42494 and *Terrisporobacter othenensis* may possess genes related to arginine biosynthesis and metabolism, making these two microbes of interest during arginine supplementation [35].

#### 3.3.2. Bacterial Metabolic Function

**Figure 6:**
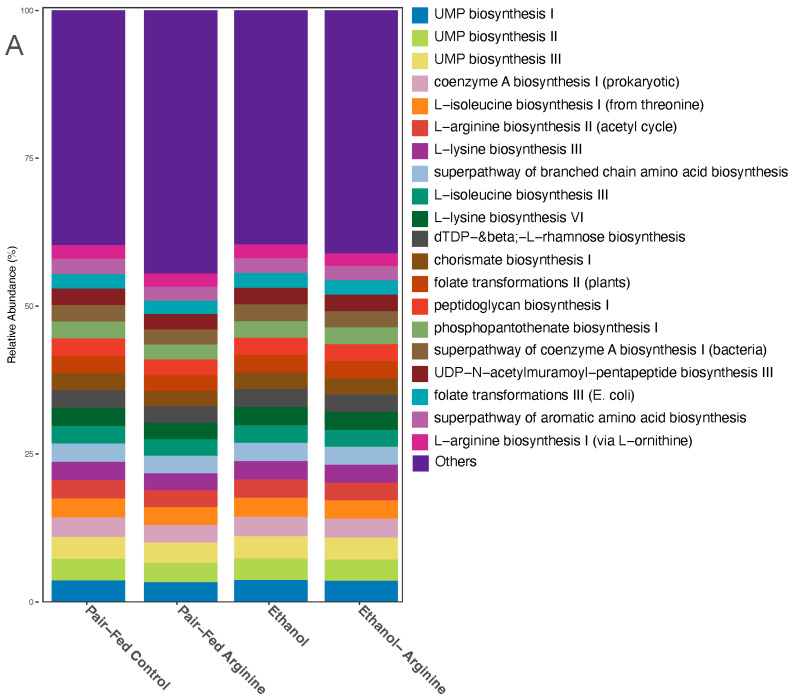

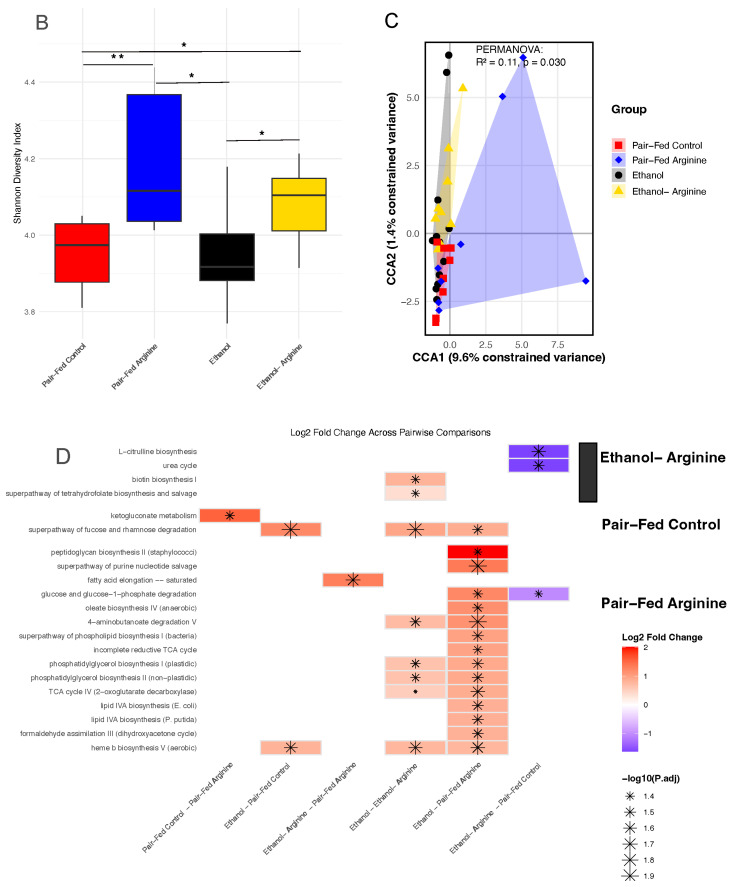
Effects of chronic ethanol feeding ± arginine on functional pathways of cecal microbiota gene expression. Following the Lieber–DeCarli chronic ethanol feeding model, the cecum was dissected, cecal contents were isolated, and gDNA was extracted using a Zymo Quick-DNA Fecal/Soil Microbe MiniPrep Kit according to the manufacturer’s instructions. (**A**) Microbial community dynamics at the functional level are shown by relative abundance calculations between treatment groups. (**B**) Alpha Diversity (Shannon Diversity Index) of functional pathways among treatment groups: ethanol, ethanol–arginine, Pair-Fed Control, and Pair-Fed Arginine, with box plots representing the interquartile range (IQR) and median values. Black asterisks indicate significant pairwise differences (* *p* < 0.05, ** *p* < 0.01) as determined by Wilcoxon rank-sum tests. (**C**) Beta-diversity is presented in the CCA plot, illustrating the first two canonical components (CC1 and CC2) that explain a significant proportion of the variance in the dataset, with samples colored by treatment group (Ethanol: Black, Ethanol-Arginine: yellow, Pair-Fed Control: red, Pair-Fed Arginine: blue) and shapes representing distinct sample groups. Shaded convex hulls enclose data points of each group, highlighting group transitions based on CCA scores. (**D**) The top microbial functional pathways between treatment groups are shown by a clustered differential abundance tile plot showing log2 fold changes (logFC) of functional pathways across pairwise comparisons, where colors denote the direction and magnitude of differential abundance (blue for decrease, red for increase, and white for no change). The size of the black points represents significance, with larger points indicating lower adjusted *p*-values (*p* < 0.05). Taxa are ordered by maximum absolute logFC for clustering effects, with facet annotations indicating corresponding groups for each taxon. All sequencing experiments represent the following number of mice per group: Pair fed-control *n* = 8, Pair-fed-arginine *n* = 8, ethanol *n* = 12, ethanol–arginine *n* = 9.

The metabolic pathways expressed by the bacterial taxa assessed with shotgun sequencing were also altered with arginine supplementation during ethanol exposure. Microbial community dynamics are shown at the functional level (Figure 6A). Shannon diversity index of the bacterial metabolic function was significantly higher in the arginine-treated groups regardless of ethanol exposure (Figure 6B). This suggests that while mice supplemented with arginine showed reduced richness and evenness (alpha-diversity) of gut bacterial taxa (Figure 5B), the metabolic genes expressed by these taxa were richer compared to mice not supplemented with arginine (Figure 6B). Per the PCoA plot, the beta-diversity analysis reveals that the feeding groups all clustered together, except for the pair-fed arginine treatment group, which had a unique bacterial gene signature compared to all other treatments (Figure 6C).

Clustered differential abundance analysis shows 15 pathways grouped with the pair-fed arginine treatment and no functional pathways cluster in the ethanol-fed mice (Figure 6D). When examining the log2 fold change pairwise comparisons between these two groups, there are 15 pathways significantly upregulated in the ethanol-fed mice compared to the pair-fed arginine group (Figure 6D). This highlights the dissimilarity between these groups. Between the pair-fed control and pair-fed arginine treatment groups, ketogluconate metabolism was the only pathway significantly increased in pair-fed control mice. Ethanol-arginine mice had a significantly increased abundance of genes related to the pathway of saturated fatty acid elongation (Figure 6D). Seven pathways were significantly upregulated in ethanol-fed mice compared to the ethanol-fed-arginine mice. These included biotin biosynthesis, phosphatidylglycerol biosynthesis, heme B biosynthesis, and the TCA cycle (IV). This demonstrates how arginine supplementation may metabolically shift the gut microbiome during chronic ethanol exposure (Figure 6D).

### 3.4. Arginine Supplementation Mitigated Ethanol-Induced Tight Junctional Protein Disruption Within the Jejunum

Communication between the gut microbiome and epithelial cells maintains intestinal homeostasis, including support for an intact gut barrier [36]. When shifts in the gut microbiome occur, damage to the gut barrier can increase intestinal permeability [37]. Arginine is critical to maintaining the normal physiology of the intestinal environment and can support the integrity of the gut barrier [38].

**Figure 7:**
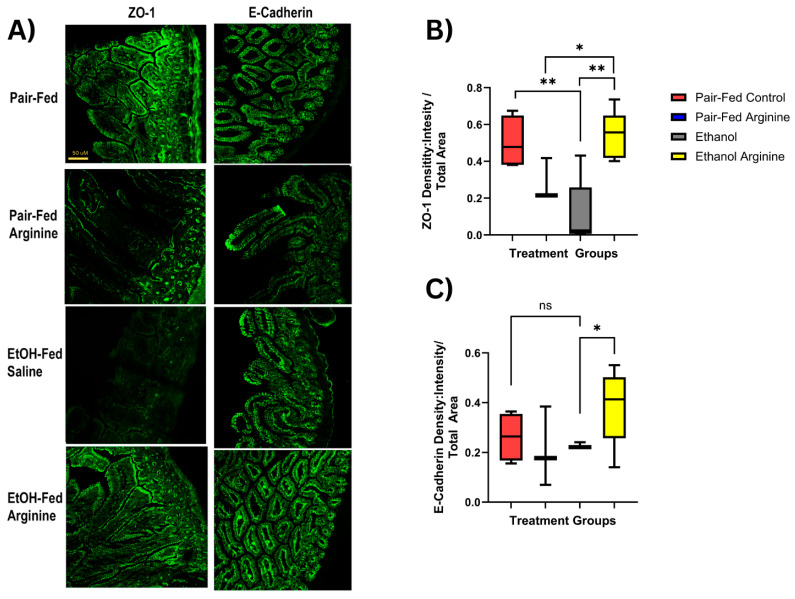
Arginine supplementation maintained tight junction protein expression in the jejunum during chronic ethanol exposure. Mice were exposed to chronic ethanol feeding following the Lieber–DeCarli diet ± ethanol ± supplemental arginine. Following euthanasia, the jejunum was dissected and used to extract RNA or was embedded in OCT for fresh frozen sectioning. 10 µm sections were cut, and staining was performed by immunohistochemistry. (**A**) Representative images are shown for ZO-1 and E-cadherin staining in the jejunum. (**B**) Density: Intensity of ZO-1 staining was acquired using ImagePro plus 7.0 software and normalized to total area. (**C**) Density: Intensity of E-Cadherin staining was acquired as above and normalized to total area. ns: not significant, * *p* < 0.05, ** *p* < 0.01. Box plots represent the following mice per group: pair fed-control *n* = 4, pair fed-arginine *n* = 4, ethanol *n* = 6, ethanol–arginine *n* = 5.

Given the ethanol-induced shifts in gut microbial composition and function observed in our chronic-ethanol-fed mice, we assessed the expression of tight junction proteins in the jejunum. We assessed for the mRNA expression of several tight junctional proteins in the jejunum (Zonula occludens-1 (ZO-1), claudin 2, and claudin-3, but did not find any changes in expression between mouse groups (Supplementary Data, Figure S2A–C). ZO-1 is a scaffolding protein that is important for the stabilization of the tight junctional protein complex [39]. We assessed the abundance and location of ZO-1 protein expression in the jejunum. Employing immunohistochemistry (IHC) analysis, we found that ZO-1 protein was localized in the apical membrane of epithelial cells (Figure 7A) and that chronic ethanol-only exposure significantly decreased protein expression compared to pair-fed control mice (*p* = 0.0059; Figure 7B). Arginine supplementation during chronic ethanol feeding significantly increased ZO-1 expression when compared to only ethanol-fed mice (*p* = 0.0013; Figure 7B).

E-Cadherin is also an important junctional protein for barrier integrity, helping to stabilize cell–cell adhesion structures [40]. When assessing for protein expression in the jejunum of only ethanol-fed mice, we did not find a statistically significant change compared to pair-fed controls. However, when comparing only ethanol-fed to ethanol-fed-arginine mice, arginine-supplemented mice had a significantly increased E-Cadherin protein expression (*p* = 0.0418; Figure 7C).

When these data are taken together, arginine supplementation may play a role in maintaining barrier integrity by supporting tight junction protein expression within the jejunum.

### 3.5. Arginine Supplementation Minimized Ethanol’s Oxidative Stress Potential in the Mouse Intestine

**Figure 8:**
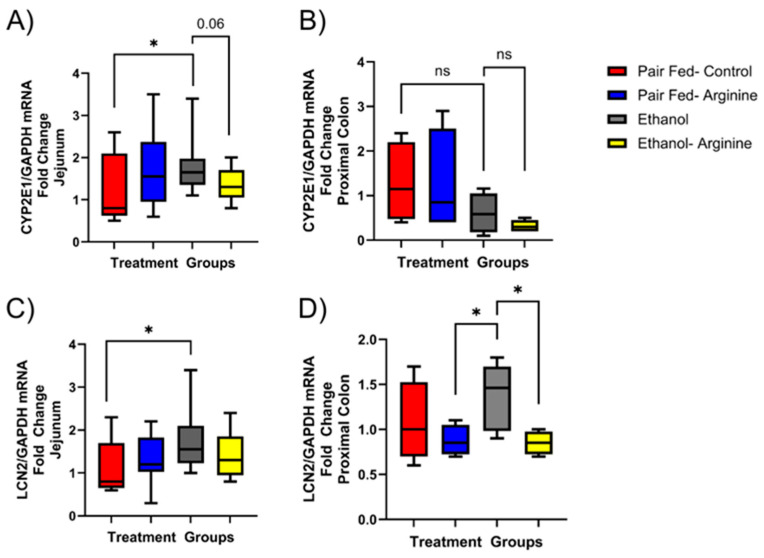
The expression of CYP2E1 and LCN2 mRNA in the intestine during chronic ethanol exposure. Mice were exposed to chronic feeding with the Lieber–DeCarli diet ± ethanol ± arginine. Following euthanasia, the jejunum and proximal colon were dissected, and RNA was extracted and used to assess for mRNA expression of CYP2E1 and LCN2 via qRT-PCR. (**A**) CYP2E1 mRNA in jejunum; (**B**) CYP2E1 mRNA expression in proximal colon; (**C**) LCN2 mRNA expression in jejunum; and (**D**) LCN2 mRNA expression in proximal colon. ns: not significant, * *p* < 0.05. For both jejunum and proximal colon mRNA, data box plots represent the following number of mice per group: pair fed-control *n* = 8, pair-fed-arginine *n* = 8, ethanol *n* = 12, ethanol–arginine *n* = 9.

Ethanol exposure is known to induce oxidative stress and intestinal inflammation. The enzyme CYP2E1 is expressed in the intestine and liver and is upregulated in response to ethanol exposure, where it converts ethanol into acetaldehyde and induces oxidative stress [41]. We found that chronic ethanol exposure significantly increased CYP2E1 mRNA expression in the jejunum compared to pair-fed controls (Figure 8A, *p* = 0.0218). In contrast, no significant changes were observed in CYP2E1 mRNA expression in mouse proximal colon tissue (Figure 8B).

Lipocalin-2 (LCN2), a marker of intestinal inflammation, helps regulate the gut microbiota by binding bacterial siderophores and preventing bacteria from using iron for their metabolism [27,42]. We found that only ethanol-fed mice had significantly increased LCN2 mRNA expression in the jejunum (Figure 8C; *p* = 0.0209) and proximal colon (Figure 8D; 0.0127). Arginine supplementation during ethanol feeding significantly reduced LCN2 mRNA expression in the proximal colon but not the jejunum compared to only ethanol-fed mice (Figure 8D).

## 4. Discussion

Ethanol exposure causes microbial imbalance, damages the gut barrier, and increases oxidative stress [1,22,37]. A disrupted intestinal environment affects the development and progression of ALD via the gut–liver axis [32]. Finding nutritional therapies to counteract ethanol-related changes in the gut–liver axis could slow or mitigate disease progression [43]. Arginine, a conditionally essential amino acid, helps manage oxidative stress [44], regulate host–microbiota co-metabolism [8], and may reduce lipid peroxidation caused by ethanol [9,19]. We demonstrate, for the first time, that arginine supplementation during chronic ethanol exposure in female mice prevented weight loss, preserved total body fat, mitigated hepatic steatosis, and reduced oxidative stress in the gut–liver axis.

Chronic ethanol consumption is known to decrease body weight, body fat and muscle mass, and muscle function [45,46]. The preservation of healthy adipose tissue is important for many physiological responses, including maintaining energy levels, insulin sensitivity, body temperature, and immune responses [47]. Our ECHO MRI data corroborate previous work showing that chronic ethanol reduced total body weight and FFM. Interestingly, arginine supplementation mitigated ethanol-induced total body weight loss and preserved total body fat mass but did not rescue FFM. The effect of L-Arginine supplementation on body composition has been investigated in various animal and human studies and has shown a favorable effect on anthropometric measures [48,49]. The preservation of FFM and improvements in blood lipid profiles during arginine supplementation in healthy men occurred when resistance exercise was added to the regimen [50]. Further investigation into arginine’s role in preserving body composition during ethanol exposure, perhaps incorporating muscle stimulation with exercise to support FFM, is warranted.

Arginine supplementation may improve lipid profiles and regulate white adipose tissue by promoting lipolysis and activating pathways involved in fatty acid oxidation [19,51,52]. In a review of relevant literature, Chen et al. [53], showed that tail-vein-injected arginine following high-fat diet-induced obesity in mice that underwent a sleeve gastrectomy reversed the expressions of β-oxidation-associated genes, reduced lipid peroxide production in the liver and inflammatory chemokines, and increased M2 macrophage markers in adipose tissue. Other studies have delivered arginine rectally in rat models, along with *Lactobacillus* strains, which have shown decreased bacterial translocation and acute liver injury [54,55]. We chose to deliver L-arginine orally in a preclinical model to evaluate its efficacy and translatability, with the aim of providing arginine as a daily oral supplement.

Previous research exploring supplemental arginine as a potential agent for lowering triglyceride levels in cardiovascular disease models indicates that changes in LPL expression could be responsible for these earlier findings [51]. LPL is the rate-limiting enzyme for TG hydrolysis, with reduced LPL levels leading to increased TG accumulation in tissues [56]. Elevated ANGPTL4 can inhibit LPL expression and lead to lipid accumulation [21,57]. Here, we show that ethanol increased the hepatic expression of ANGPTL4 and decreased LPL, and this was linked to elevated liver TG. Interestingly, arginine supplementation mitigated ANGPTL4 levels, which rescued LPL and liver TG during ethanol exposure. Together, this shows arginine’s potential to promote lipolysis in the liver and prevent hepatic steatosis induced by ethanol exposure. Further research investigating arginine’s effect on hepatic de novo lipogenesis and fatty acid β-oxidation during ethanol exposure is warranted.

In addition to the role of ANGPTL4 in mediating lipid metabolism, studies show a role for hepatic ANGPTL4 in triggering the lipotoxic accumulation of fatty acids and producing reactive oxygen species (ROS) [58,59]. When assessing 4-HNE adducts in liver tissue, arginine supplementation during ethanol feeding significantly reduced the expression of these lipid peroxidation by-products compared to only ethanol-fed mice. 4-HNE protein adducts in the liver have been shown to target specific proteins, especially those involved in catalytic, transferase, and hydrolase activities [60]. Our data show notable changes in both the overall levels of 4-HNE and the presence of a 4-HNE adduct at the 25 kDa molecular weight. Our current method cannot accurately identify this protein; however, it is important to note that many proteins involved in hepatic steatosis development have similar molecular weights. Further work is needed to identify proteins that may be modified by 4-HNE in a model of chronic ethanol exposure.

Disruption in host–microbe interactions along the gut–liver axis has been observed in a variety of liver diseases, including ALD [61,62]. The gut microbiome harbors microbial members that are beneficial for host metabolism and digestion. Chronic ethanol exposure leads to gut bacterial dysbiosis, a dysfunction in microbial composition and function [62]. Studies in germ-free mice have shown that the gut microbiome may regulate ANGPTL4 expression and that shifts in microbial taxa can also control lipid peroxidation [63,64]. We hypothesized that arginine supplementation may influence lipid profiles during chronic ethanol exposure by altering the structure and function of the gut microbiota. Our beta diversity data reveal unique bacterial compositions between treatment groups. Ethanol-fed mice showed significantly higher levels of Firmicutes phylum taxa *Clostridium culturomicium*, *Lachnospiraceae bacterium* MD329, and *Enterococcus faecalis* compared to pair-fed controls. Firmicutes expansion has previously been linked to high-fat feeding models, and these ethanol-induced taxa can participate in pathways related to fatty acid synthesis and metabolism [63,65]. When comparing the cecal microbiota of only ethanol-fed mice and ethanol-fed + arginine mice, we also observe changes in members of the Firmicutes phylum. Mice fed ethanol + arginine showed a decreased abundance of *Terrisporobacter othenensis*, *Lachnospiraceae Bacterium*_28, and *Enterococcus faecium* compared to those only fed ethanol. *T. othenensis* is a Gram-positive microbe initially isolated from blood and identified as a cause of sepsis [66]. Genomic analysis of this bacterium and related species within the *Terrisporobacter* genus suggests that it possesses various virulence factors, including genes related to iron uptake, like those found in *Vibrio* and *Haemophilus* [67]. *E. faecium* is another Gram-positive microbe widely regarded as an opportunistic pathogen responsible for nosocomial infections [68]. Arginine supplementation may influence the virulence and growth of *E. faecium* and other *Enterococcus* species [69]. Overall, these data suggest chronic ethanol feeding may shift the microbial composition to favor fatty acid production and that arginine supplementation may reduce the abundance of potentially pathogenic gut microbes during chronic ethanol exposure.

Through shotgun metagenomic sequencing, we were able to analyze changes in the metabolic pathways and overall functional potential of the cecal microbiota. Our data identified seven metabolic pathways that were significantly different between only ethanol-fed and ethanol-fed arginine microbiota. These included biotin biosynthesis I, Phosphatidylglycerol biosynthesis I and II, heme B biosynthesis, and the TCA cycle (pathway IV), all of which have implications for lipid metabolism and oxidative damage.

Phosphatidylglycerol (PG) is a structural lipid and a key component of bacterial membranes. Increased PG biosynthesis can enhance bacterial survival by reinforcing membrane integrity [70]. Lipidomic studies have identified links between PGs and gut microbiota imbalance, showing bacterial PG production was associated with markers of intestinal inflammation and increased lipolysis in host adipose tissue [71].

The heme B pathway, when elevated, may promote bacterial colonization, overgrowth, and increase iron-related oxidative stress in the intestine [72]. We found heme B biosynthesis was significantly higher in only ethanol-fed mice compared to pair-fed control, pair-fed arginine, and ethanol-fed arginine mice. These findings suggest that arginine might help reduce the heme B biosynthesis pathway induced by chronic ethanol exposure. Microbial heme production is linked to intestinal inflammation and disrupted iron homeostasis between the host and microbiota [73]. Together, these dysregulated pathways can trigger ferroptosis in both hepatic and intestinal cells. Ferroptosis is a form of cell death dependent on iron-induced lipid peroxidation. Recent literature has indicated that ferroptosis-mediated cell death may contribute to the development and progression of ALD [74].

Gut dysbiosis and the overabundance of pathogenic bacteria contribute to an impaired gut barrier, which is a prerequisite for an altered gut–liver axis. The gut barrier protects against potentially toxic metabolites, bacteria, and their antigens exiting the gut lumen and entering the portal circulation [37]. The intestinal epithelial barrier is tightly regulated by a tight junctional protein complex. We assessed for the expression of tight junctional proteins in the jejunum and showed that, compared to only ethanol-fed mice, arginine supplementation rescued the depleted expression of ZO-1 and E-cadherin. ZO-1 is a crucial scaffolding protein that anchors transmembrane proteins to the cytoskeleton to maintain barrier integrity [37]. E-cadherin is an essential transmembrane protein and the main component of adherens junctions, where it maintains structural integrity and cell-to-cell adhesion and regulates tight junctions [40]. Together, these data support arginine supplementation’s beneficial effect on the small intestinal epithelial barrier during ethanol exposure.

Chronic ethanol exposure induces oxidative stress in the liver as well as the intestine, where CYP2E1 is known to be induced in intestinal epithelial cells in the presence of ethanol [75]. CYP2E1 activity produces reactive oxygen species that cause damage to epithelial integrity and can enter enterohepatic circulation to influence the progression of ALD [24,41,75]. In our chronic ethanol feeding model, we found that intestinal CYP2E1 mRNA expression was increased in the jejunum but not the proximal colon of only ethanol-fed mice compared to pair-fed controls. This is likely because ethanol is primarily absorbed in the stomach and jejunum, and ethanol levels in the colon may not be high enough to induce CYP2E1 expression.

Intestinal LCN2 is a recognized biomarker for intestinal inflammation and interacts with bacterial heme and iron siderophores [27,42]. LCN2 gene expression may also serve as a key regulator in ferroptosis and facilitate lipid peroxidation [42]. In both the jejunum and proximal colon, only ethanol-fed mice exhibited significantly higher LCN2 mRNA levels compared to pair-fed controls. This highlights the pro-oxidant effect of chronic ethanol exposure across the intestinal environment and aligns with emerging research emphasizing that iron homeostasis and ferroptosis are crucial mechanisms in the development and progression of ALD [76,77,78]. In our model, ethanol-induced LCN2 mRNA expression was significantly reduced by ethanol-fed + arginine treatment in the proximal colon but not in the jejunum. These findings suggest that further investigation is needed to better understand the region-specific mechanisms through which arginine supplementation could mitigate ethanol-induced intestinal oxidative damage.

The data presented here suggest a potential role for L-arginine supplementation in mitigating ethanol-induced hepatic steatosis, the first stages of ALD. The current study is limited in that the findings are associative and do not identify underlying mechanisms that cause the changes identified. Although our pre-clinical model of chronic ethanol feeding shows promise that arginine oral supplementation may beneficially impact hepatic triglyceride accumulation and oxidative stress during chronic ethanol feeding, further studies are needed to determine if these preliminary effects can be realized during clinical human conditions of chronic ethanol exposure and progression of ALD. It would also be interesting to determine if arginine supplementation can reverse hepatic steatosis induced by chronic ethanol exposure. However, our findings are a first step toward determining if arginine supplementation may be a potential therapeutic approach for supporting the gut–liver axis disrupted by chronic ethanol exposure.

## Figures and Tables

**Table 1:** Primer sequences used for RT-qPCR.

Primer Target (Abbreviation)	Forward Sequence	Reverse Sequence	Primary NCBI Accession Number
Cytochrome P450 2E1 (CYP2E1)	GGG ACA TTC CTG TGT TCC AG	GGC CTC ATT ACC CTG TTT CC	NM_021282.3
Lipocalin 2 (LCN2)	TGG CCC TGA GTG TCA TGT G	CTC TTG TAG CTC ATA GAT GGT GC	NM_008491
Glyceraldehyde-3-Phosphate Dehydrogenase (GAPDH)	AGG TCG GTG TGA ACG GAT TTG	TGT AGA CCA TGT AGT TGA GGT CA	NM_001289726
Zonula Occludens -1 (ZO-1)	TGG GAA CAG CAC ACA GTG AC	GCT GGC CCT CCT TTT AAC AC	NM_009386
Claudin-2 (CLDN2)	CAA CTG GTG GGC TAC ATC CTA	CCC TTG GAA AAG CCA ACC G	NM_016675.1
Claudin-3 (CLDN3)	CCT CAT CGT GGT GTC CAT CC	CGT CTC GTC TTG TAC GCA GT	NM_001306

**Table 2:** Antibodies used for Western Blotting.

Antibody Name (Abbreviation)	Supplier	Catalog Number	Location
Cytochrome P450 2E1 (CYP2E1)	Abcam	ab28146	Cambridge, MA, USA
4-hydroxy-2-nonenal (4-HNE)	Alpha Diagnostic	HNE11S	San Antonio, TX, USA
Angiopoietin-like 4 (ANGPTL4)	Abcam	ab2920	Cambridge, MA, USA
Beta-Actin (β-Actin)	Cell Signaling	4967S	Danvers, MA, USA
Heat Shock Cognate 70 kDa protein (HSC70)	Santa Cruz Biotech	sc7298	Dallas, TX, USA
Lipoprotein Lipase (LPL)	GeneTex	GTX101125	Irvine, CA, USA
Poly (ADP-ribose) Polymerase-1 (PARP1)	Cell Signaling	9542S	Danvers, MA, USA
Lipocalin-2 (LCN2)	R&D Systems	AF1757	Minneapolis, MN, USA

## Data Availability

Data supporting the reported results can be requested by writing to the corresponding author, and the microbiome data are openly available in NCBI at PRJNA1439325.

## Supplementary Materials

The following supporting information can be downloaded at https://www.mdpi.com/article/10.3390/antiox15050537/s1, Table S1: Nutritional Facts of the Lieber DeCarli Liquid Diet. Figure S1: Chronic ethanol exposure ± arginine supplementation does not change hepatic LCN2 expression. Figure S2: Chronic ethanol exposure ± arginine supplementation does not change hepatic PARP1 expression. Figure S3: Chronic ethanol feeding ± supplemental arginine does not change mRNA expression of jejunum tight junctions.

## Abbreviations

The following abbreviations are used in this manuscript:

ALD	Alcohol-associated liver disease
FFM	Fat-free mass
TG	Triglyceride
GPO	Glycerol phosphate oxidase
CT	Comparative Threshold
GAPDH	Glyceraldehyde 3-phosphate
qRT-PCR	Real-Time Quantitative Polymerase Chain Reaction
CYP2E1	Cytochrome P50 2E1
Lcn2	Lipocalin 2
4-HNE	4-hydroxy-2-nonenal
ANGPTL4	Angiopoietin-like 4
ROUT	Robust nonlinear regression method
Hsc70	Heat Shock Cognate 70 kDa protein
TBW	Total body weight
ROS	Reactive oxygen species
PG	Phosphatidylglycerol
LPL	Lipoprotein lipase
ZO-1	Zonula Occludens 1
CLDN2	Claudin-2
CLDN3	Claudin-3
PARP1	Poly (ADP-Ribose) Polymerase 1
GI	Gastrointestinal
logFC	Log Fold Change
IQR	Interquartile range
IHC	Immunohistochemistry
OCT	Optimal cutting temperature
