# Supplementary material for: Role of L-Arginine in the Gut–Liver Axis of Female Mice: Mediating Ethanol’s Alterations in Hepatic Steatosis and Oxidative Stress
Source: Antioxidants (Basel). 2026 Apr 24;15(5):537. doi: 10.3390/antiox15050537 (PMC13203133; doi:10.3390/antiox15050537)

Supplementary Data: Role of L-Arginine in the Gut-Liver Axis of Female Mice: Mediating Ethanol's Alterations in Hepatic Steatosis and Oxidative Stress.

Table S1: Nutritional Facts of the Lieber DeCarli Liquid Diet. Table shows nutrients per liter of liquid diet mix used in chronic-ethanol feeding model outlined in section 2.1. Adapted from Bio-Serv.com Product #F1259 (BioServ, NJ)

Nutrient	Content per Liter
Carbohydrate	490 kcals/L
Protein	151 kcals/L
Fat	359 kcals/L
L-Arginine	1.3gm/L

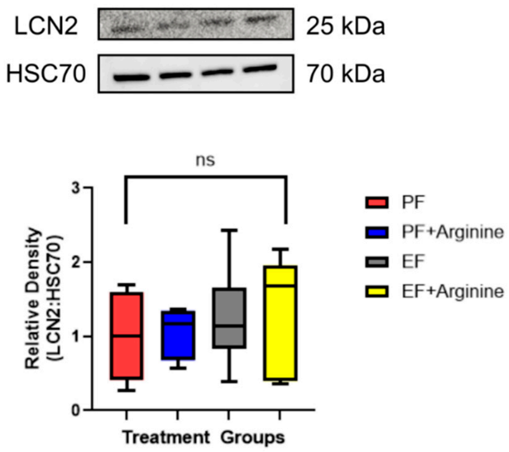

Figure S1: Chronic ethanol exposure ± arginine supplementation does not change hepatic LCN2 expression. Following the 25-day Lieber DeCarli chronic ethanol feeding model, as described in Fig.1, mice were euthanized, and the liver was dissected and used to assess protein expression of lipocalin-2 (LCN2). Bands for LPL were visualized at 25 kDa and normalized to the expression of HSC70. Representative bands shown. Densitometry analysis of LCN2: HSC70 protein expression. Box plots represent the following number of animals per group: Pair-fed control n= 8, pair fed-arginine n= 4, ethanol n= 12, ethanol-arginine n=5. NS: not significant. PF: pair-fed; EF: ethanol-fed.

Figure S2: Chronic ethanol exposure ± arginine supplementation does not change hepatic PARP1 expression. Following the 25-day Lieber DeCarli chronic ethanol feeding model, as described in Fig.1, mice were euthanized, and liver was dissected and used to assess protein expression of PARP1. Bands for PARP1 were visualized at both 89 kDa and 116 kDa. HSC70 was used to normalize PARP1 expression. A) Densitometry analysis was assessed with both PARP1 bands combined relative to HSC70. B) PARP1 bands visualized at 89 kDa were analyzed independently and normalized to HSC70 expression. C) PARP1 bands visualized at 116 kDa were analyzed independently and normalized to HSC70 expression. Box plots represent the following number of mice per group= pair fed-control n= 4, pair fed-arginine n= 4, ethanol n= 6, ethanol-arginine n= 5. NS= no significance. PF: pair-fed; PF-A: pair-fed-arginine; EF: ethanol-fed only; EF-A: ethanol-fed-arginine.

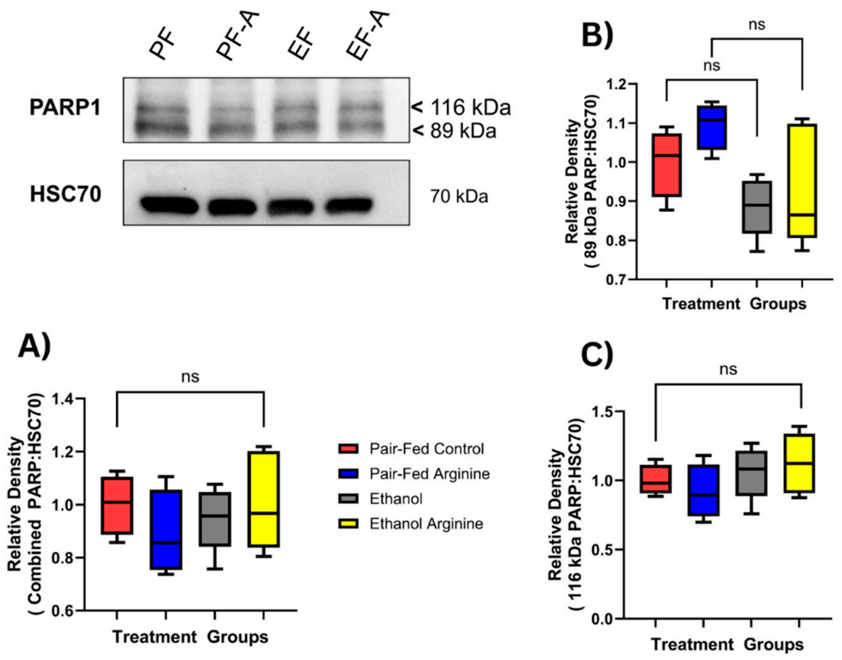

Figure S3: Chronic ethanol feeding ± supplemental arginine does not change mRNA expression of jejunum tight junctions. Mice were exposed to chronic feeding with the Lieber-DeCarli diet ± ethanol ± arginine as outlined in Figure 1. Following euthanasia, jejunum was dissected, RNA was extracted, and used to assess for mRNA expression of tight junctional proteins using qRT-PCR. A) ZO-1 mRNA: GAPDH expression is shown as a fold change normalized to pair-fed controls; B) Claudin-2 mRNA: GAPDH expression; C) Claudin-3 mRNA: GAPDH expression in the proximal colon. Bar graphs represent n=6-12 mice per group. NS: not significant. Box plots for all mRNA data represent the following number of mice per treatment group= Pair fed-control n= 8, Pair-fed-arginine n=8, Ethanol n= 12, Ethanol- Arginine n= 9.

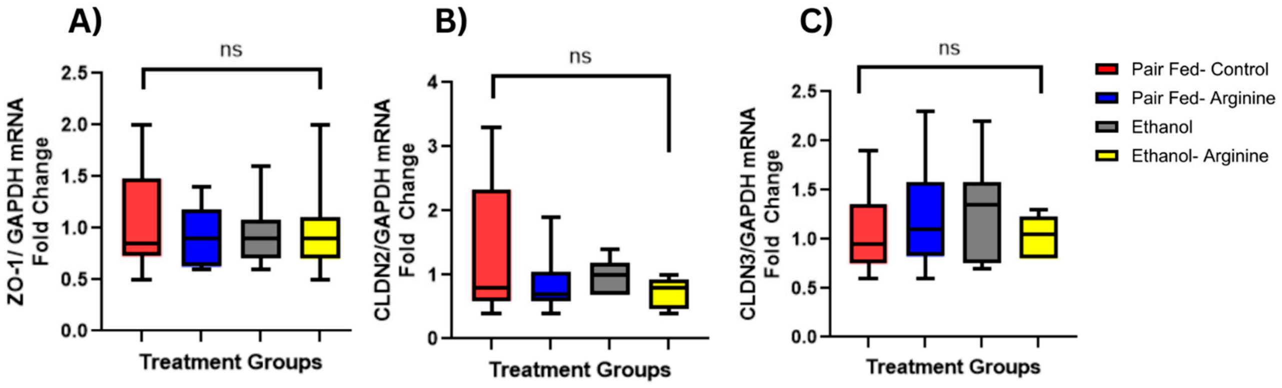

Supplement: Supplementary file 1 [file antioxidants-15-00537-s001.zip › antioxidants-4228610-supplementary.pdf]
